# Inhibition of mucus secretion by niclosamide and benzbromarone in airways and intestine

**DOI:** 10.1038/s41598-024-51397-w

**Published:** 2024-01-17

**Authors:** Jiraporn Ousingsawat, Raquel Centeio, Nicole Reyne, Alexandra McCarron, Patricia Cmielewski, Rainer Schreiber, Gabriella diStefano, Dorothee Römermann, Ursula Seidler, Martin Donnelley, Karl Kunzelmann

**Affiliations:** 1 Physiological Institute, University of Regensburg, University Street 31, 93053 Regensburg, Germany; 2 Robinson Research Institute and Adelaide Medical School, University of Adelaide, Adelaide, SA Australia; 3 Department of Gastroenterology, Hannover Medical School, 30625 Hannover, Germany

**Keywords:** Respiration, Gastrointestinal diseases, Respiratory tract diseases

## Abstract

The Ca^2+^ activated Cl^−^ channel TMEM16A (anoctamin 1; ANO1) is expressed in secretory epithelial cells of airways and intestine. Previous studies provided evidence for a role of ANO1 in mucus secretion. In the present study we investigated the effects of the two ANO1-inhibitors niclosamide (Niclo) and benzbromarone (Benz) in vitro and in vivo in mouse models for cystic fibrosis (CF) and asthma. In human CF airway epithelial cells (CFBE), Ca^2+^ increase and activation of ANO1 by adenosine triphosphate (ATP) or ionomycin was strongly inhibited by 200 nM Niclo and 1 µM Benz. In asthmatic mice airway mucus secretion was inhibited by intratracheal instillation of Niclo or Benz. In homozygous F508del-cftr mice, intestinal mucus secretion and infiltration by CD45-positive cells was inhibited by intraperitoneal injection of Niclo (13 mg/kg/day for 7 days). In homozygous F508del-cftr rats intestinal mucus secretion was inhibited by oral application of Benz (5 mg/kg/day for 60 days). Taken together, well tolerated therapeutic concentrations of niclosamide and benzbromarone corresponding to plasma levels of treated patients, inhibit ANO1 and intracellular Ca^2+^ signals and may therefore be useful in inhibiting mucus hypersecretion and mucus obstruction in airways and intestine of patients suffering from asthma and CF, respectively.

## Introduction

Expression of the Ca^2+^ activated Cl^−^ channel (CaCC) TMEM16A (anoctamin 1; ANO1) is upregulated in inflammatory airway diseases such as cystic fibrosis (CF) or asthma^1–4^. Upregulation of ANO1 correlates with enhanced mucus secretion observed under inflammatory conditions^5–9^. A number of ANO1 blockers, including niclosamide and benzbromarone were shown to inhibit mucus secretion and bronchodilation, respectively^1,10–13^. In contrast, activators of ANO1 such as Eact and brevenal caused mucus release and bronchoconstriction^4,13,14^. Moreover, enhanced activity of ANO1 was found to correlate with vasoconstriction and pulmonary hypertension^15–18^. Taken together these studies suggest that inhibition of ANO1 could be useful in treating lung diseases such as CF and asthma.

ANO1 is inhibited by a diversity of compounds, including niclosamide, benzbromarone, niflumic acid, 5-nitro-2-(3-phenylpropylamino)-benzoate (NPPB), or CaCCinh-AO1^19,20^. These structurally unrelated compounds are mostly lipophilic and probably bind to the lipophilic drug binding pocket present in anoctamin channels and scramblases, respectively^21^. High-throughput screening identified the anthelmintic drug niclosamide as a potent ANO1-inhibitor^13^. It was shown to strongly bronchodilate mouse and human airways in vitro, while subsequent studies demonstrated inhibition of airway mucus and cytokine secretion in asthmatic mice in vivo^13,22,23^, ex vivo^22^ and in vitro^8^. Niclosamide strongly reduces mucin production and inhibits exocytosis of mucus in airways and intestine by blocking ANO1 and ANO6^4,7,22,24^. Drug screening also identified the non-steroidal anti-inflammatory compound benzbromarone as inhibitor of ANO1^1^. Benzbromarone similarly inhibits airway mucus secretion and bronchoconstriction, and improves the asthmatic phenotype in mice^1,2,25^.

It has been argued that niclosamide and benzbromarone may inhibit activation of ANO1 channels only indirectly, i.e. not by binding to ANO1 but by causing an off-target effect that strongly alters intracellular Ca^2+^ ([Ca^2+^]_i_) signals. It was concluded that these off-target effects may preclude the use in patients^26,27^. It should be noted, however, that ANO1 itself controls both basal and agonist-induced rise in [Ca^2+^]_i_: both inhibition and knockdown of ANO1 attenuated secretagogue-induced [Ca^2+^]_i_ increase, while overexpression and activation of ANO1 demonstrated opposite effects^4,28^. Moreover, inhibitors of ANO1 such as niclosamide and benzbromarone are often used experimentally at excessive concentrations, enhancing the likelihood for off-target effects. In the present study we report that low concentrations of these inhibitors typically found in treated patients block ion currents produced by ANO1, and inhibit intracellular Ca^2+^ signals. We examined tolerability and effectiveness of these drugs which may help to inform future clinical trials. Inhibition of mucus secretion by niclosamide and benzbromarone is shown in different animal models for asthma and cystic fibrosis. Recent data are discussed on tolerability and usefulness of both drugs.

## Results

### Niclosamide and benzbromarone inhibit endogenous ANO1 in airway epithelial cells at therapeutic concentrations

**Figure 1:**
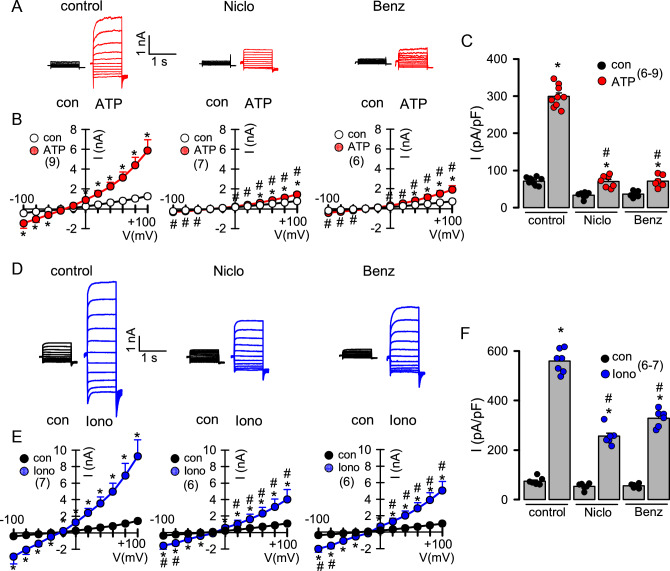
Inhibition of ANO1 by niclosamide and benzbromarone in CFBE airway epithelial cells. (**A**) Current overlays showing activation of whole cell currents by ATP (10 µM), which was inhibited by niclosamide (Niclo; 200 nM) and benzbromarone (Benz; 1 µM). (**B**,**C**) Corresponding current/voltage (I/V) relationships and current densities in the absence (n = 9) or presence of Niclo (n = 7) or Benz (n = 6). (**D**,**E**) Whole cell current overlays, I/V relationships and current densities of ANO1 activated by 1 µM ionomycin in the absence (n = 7) or presence of Niclo (n = 6) and Benz (n = 6). Mean ± SEM (number of experiments). *significant activation by ATP and ionomycin, respectively (p < 0.05; paired t-test). ^#^significant inhibition by Niclo and Benz (p < 0.05; ANOVA & post hoc Bonferroni).

Studies reported variable plasma peak concentrations for niclosamide (Niclo; 700–18,000 nM)^29,30^ and benzbromarone (Benz; ~ 6000 nM)^31^, which dropped to clearly lower concentrations within the following 24 h. In the present study, we examined the effects of Niclo and Benz at concentration of 200 nM and 1000 nM respectively, in order to stay within the therapeutic concentration range. At these concentrations ANO1 whole cell currents activated by the purinergic ligand ATP (10 µM) in CFBE14o- airway epithelial cells were potently inhibited (Fig. 1A–C). The experiments were performed in the presence of 100 nM TRAM-34 to avoid potential activation of Ca^2+^ activated K^+^ channels^32^. Niclo and Benz are likely to inhibit ANO1 currents directly by binding to its lipophilic binding pocket^21^ and probably also by lowering agonist-induced Ca^2+^ release and/or Ca^2+^ influx^25–28^. We therefore activated ANO1 also by increasing [Ca^2+^]_i_ directly using the Ca^2+^ ionophore ionomycin (1 µM). Under these conditions both Niclo and Benz still inhibited ionomycin-activated ANO1 currents, albeit inhibition was less pronounced (Fig. 1D–F). Moreover, in additional experiments Niclo and Benz were applied in the presence of 1 µM Ca^2+^ in the patch pipette filling solution. Under these conditions the ANO1 current (measured at a clamp voltage of + 100 mV) was significantly (p < 0.05; paired t-tests) inhibited from 8.4 ± 1.1 nA (− Niclo) to 5.1 ± 0.8 nA (+ 5 µM Niclo; n = 4), and from 8.0 ± 0.9 nA (− Benz) to 4.2 ± 0.9 nA (+ 10 µM Benz; n = 4). The data confirm that both blockers inhibit ANO1 in the presence of high cytosolic Ca^2+^ concentration and at concentrations found as plasma concentrations in patients.

### Niclosamide and benzbromarone inhibit intracellular Ca^2+^ signals, but also inhibit ANO1 independent of changes in the cytosolic Ca^2+^ concentration

**Figure 2:**
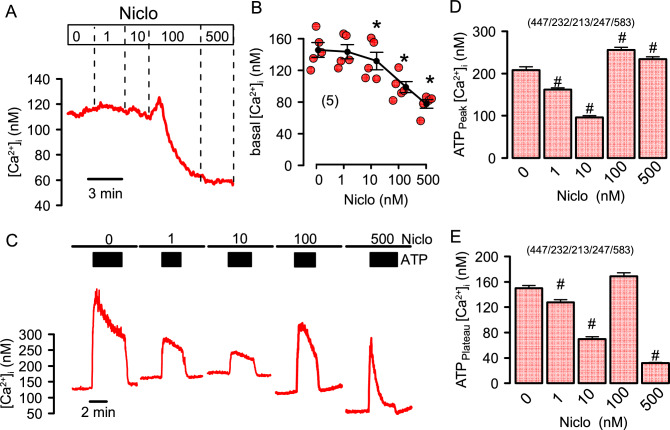
Effects of niclosamide on intracellular Ca^2+^ in CFBE airway epithelial cells. (**A**) Changes of basal intracellular Ca^2+^ concentrations [Ca^2+^]_i_ in CFBE airway epithelial cells by consecutive application of niclosamide (Niclo) at concentrations ranging from 0 to 500 nM. (**B**) Summary of basal [Ca^2+^]_i_ changes induced by application of different concentrations of Niclo (n = 5 for all). (**C**) Effect of 0 (n = 447), 1 (n = 232), 10 (n = 213), 100 (n = 247), and 500 (n = 583) nM Niclo on ATP-induced (10 µM) increase in [Ca^2+^]_i_. Cells were preincubated with Niclo for 5 min and was present throughout the experiment. (**D**) Summary of the ATP-induced peak [Ca^2+^]_i_ (ER Ca^2+^ store release) in the presence of increasing concentrations of Niclo. (**E**) Summary of the ATP-induced plateau [Ca^2+^]_i_ (SOCE). Mean ± SEM (number of cells analysed). *significant inhibition by Niclo (p < 0.05; ANOVA & post hoc Bonferroni).

**Figure 3:**
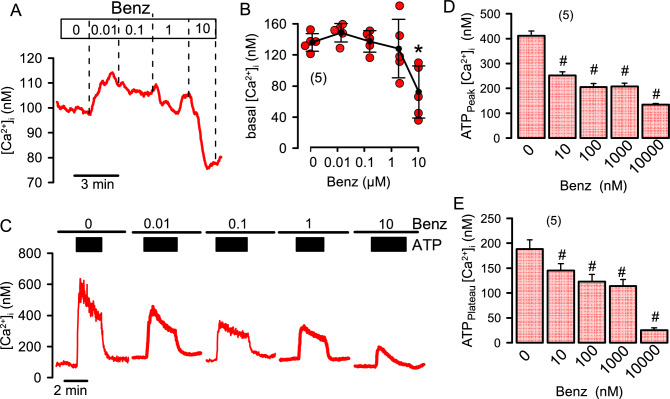
Effects of benzbromarone on intracellular Ca^2+^ in CFBE airway epithelial cells. (**A**) Change of basal intracellular [Ca^2+^]_i_ in CFBE airway epithelial cells by consecutive application of benzbromarone (Benz) at concentrations ranging from 10 to 10,000 nM (n = 5 for all). (**B**) Summary for [Ca^2+^]_i_ changes induced by application of different concentrations of Benz (n = 5 for all). (**C**) Effect of different concentrations of Benz on ATP-induced (10 µM) increase in [Ca^2+^]_i_. Cells were preincubated with Benz for 5 min which was present throughout the experiment. (**D**) Summary of ATP-induced peak [Ca^2+^]_i_ increase in the presence of increasing concentrations of Benz (n = 5 for all). (**E**) Summary of the ATP-induced plateau [Ca^2+^]_i_ (SOCE). Mean ± SEM (number of cells analysed). *Significant inhibition by Benz (p < 0.05; ANOVA: & post hoc Bonferroni).

We measured the effects of Niclo and Benz on [Ca^2+^]_i_ in CFBE cells loaded with the Ca^2+^ sensor Fura2. Niclo concentrations ≥ 100 nM induced a brief and transient Ca^2+^ increase, which was followed by a sustained decrease in basal [Ca^2+^]_i_ (Fig. 2A,B). Ca^2+^ increase by the purinergic agonist ATP was assessed in the presence of increasing concentration of Niclo. ATP (10 µM) induced a sudden release of Ca^2+^ from the endoplasmic reticulum (ER) (peak Ca^2+^), which was followed by a Ca^2+^ influx (store operated Ca^2+^ entry; SOCE) (plateau Ca^2+^). At low (1 and 10 nM) concentrations, Niclo inhibited ATP-induced peak Ca^2+^ and proportionally inhibited plateau Ca^2+^ (Fig. 2C–E). In contrast, 100 and 500 nM Niclo increased peak Ca^2+^, while SOCE was strongly inhibited at 500 nM. These data suggest complex and concentration-dependent effects of Niclo on basal [Ca^2+^]_i_ and ATP-induced Ca^2+^ increase. Benz inhibited basal [Ca^2+^]_i_ only at 10 µM (Fig. 3A,B). However, it attenuated ATP-induced peak and plateau Ca^2+^ in a concentration-dependent manner (Fig. 3C–E). Taken together, inhibitory effects of Niclo and Benz on [Ca^2+^]_i_ occur at concentrations measured in the plasma of treated patients, and can be explained by concentration-dependent inhibition of Ca^2+^ uptake into the store by inhibition of the sarcoplasmic endoplasmic reticulum ATPase (SERCA) and/or by blockade of Orai1-dependent Ca^2+^ entry (SOCE)^25^. The data provide evidence that both compounds inhibit ANO1 directly (by binding to ANO1) and indirectly (by lowering cytosolic Ca^2+^).

We previously demonstrated that ANO1 largely augments release of Ca^2+^ from the ER-store, while knockout or inhibition of ANO1 attenuates store release^7,22,28,33^. Moreover, ANO1 directly interacts with inositol trisphosphate receptors (IP_3_R) and therefore possibly controls the activity of IP_3_R^28,34^. Notably, after knockdown of ANO1, niclosamide no longer inhibited Ca^2+^ store release^25^. Therefore, Niclo and probably other ANO1 inhibitors like Benz affect intracellular Ca^2+^ signals at least partially through inhibition of ANO1. Finally, ANO1 and ANO6 functionally interact with Ca^2+^ influx pathways such as TRPV1 or TRPV4, which probably shape intracellular Ca^2+^ signals^35–38^. Taken together, both niclosamide and benzbromarone show pronounced inhibitory effects on intracellular Ca^2+^ signals, which interferes with activation of ANO1. Nevertheless, niclosamide and benzbromarone also inhibit ANO1 directly and independent of changes in the cytosolic Ca^2+^ concentration.

### Inhibition of mucus secretion by niclosamide and benzbromarone in mouse asthmatic airways

**Figure 4:**
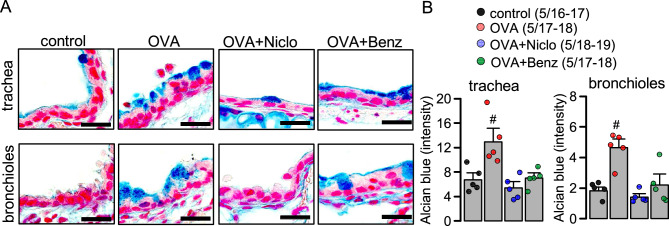
Inhibition of mucus secretion by niclosamide and benzbromarone in airways of asthmatic mice. (**A**) Alcian blue staining of mucus in trachea and bronchioles from control animals and OVA-treated asthmatic mice stained by haematoxylin & eosin. Intratracheal instillation of 30 µM niclosamide (Niclo) or 30 µM benzbromarone (Benz) dissolved in 100 µl saline, or 100 µl saline only (OVA) for 5 consecutive days. Bars = 20 µm. (**B**) Summary of alcian blue staining of airway mucus (blue colour intensity per section) in trachea (n = 5 animals/n = 16–19 sections analysed for each animal) and bronchioles (n = 5 animals/n = 16–19 sections analysed for each animal). Mean ± SEM. ^#^Significant difference when compared to control (p < 0.05; ANOVA & post hoc Bonferroni).

Airway mucus plugging is common in asthma^39^. We compared the ability of Niclo and Benz to inhibit mucus secretion in asthmatic mice in vivo. Niclo or Benz (both 30 µM), dissolved in 100 µl saline, or 100 µl saline only (OVA; control), were applied by intratracheal instillation to ovalbumin-treated asthmatic mice over five consecutive days. Afterwards, animals were humanely killed and airways were analysed for mucus secretion using alcian blue staining. Ovalbumin increased mucus production in trachea and bronchi due to goblet cell metaplasia (Fig. 4). Benz and especially Niclo strongly attenuated mucus production in trachea and particularly in bronchioles of OVA mice, to a level present in control mice (Fig. 4). To our knowledge this is the first study in which benzbromarone was applied to inflamed (asthmatic) mouse airways in vivo. It shows muco-inhibitory effects comparable to niclosamide. We conclude that both drugs may be useful therapeutics for the treatment of airway mucus hypersecretion, which is commonly observed in asthma and cystic fibrosis.

### Inhibition of intestinal mucus secretion by niclosamide in F508del-cftr/F508del-cftr mice

**Figure 5:**
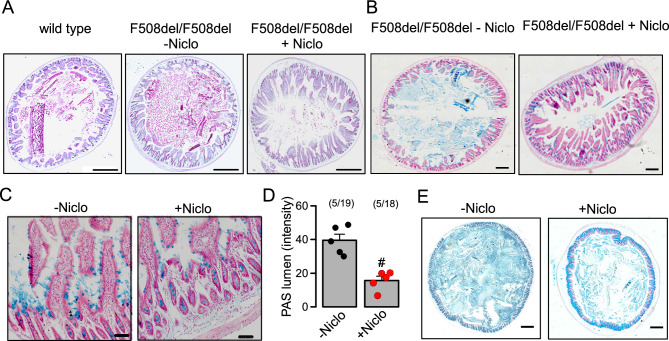
Inhibition of mucus secretion by niclosamide in intestine of homozygous F508del-cftr mice. (**A**) PAS staining of mucus in jejunum of a wild type mouse, and homozygous F508del-cftr mice treated by intraperitoneal injection of corn oil only (−Niclo) or niclosamide (13 mg/kg/d dissolved in corn oil) (+Niclo) for 7 consecutive days. Tissue staining by haematoxylin & eosin. Bars = 400 µm. (**B**,**C**) Alcian blue staining of mucus in jejunum at lower and higher magnification. Bars = 400 µm and 50 µm, respectively. (**D**) Summary of alcian blue staining (blue colour intensity per section) (n = 5 animals/n = 18–19 sections analysed for each animal). (**E**) Alcian blue staining of mucus in colon of F508del-cftr/F508del-cftr mice. Mean ± SEM (number of animals/number of sections analysed). ^#^significant difference when compared to −Niclo (p < 0.05; unpaired t-test).

Mucus hypersecretion resulting in airway plugging is also observed in human CF airways^40^. In contrast, rodent CF models develop only a mild lung phenotype, but frequently demonstrate intestinal obstructions^41–43^. Because we previously showed a role of ANO1 for intestinal mucus secretion^7,22^, we examined the effects of Niclo and Benz on intestinal mucus production in CF mice and rats. Mice homozygous for the most frequent cystic fibrosis transmembrane conductance regulator (cftr) mutation F508del, demonstrate intestinal mucus accumulation, leading to bacterial overgrowth, inflammation, slower intestinal transit and lethal obstructions^41,44^. We hypothesized that inhibition of ANO1 by Niclo may attenuate excessive intestinal mucus secretion. To that end, F508del/F508del-cftr mice were treated by intraperitoneal injections of niclosamide (13 mg/kg/d dissolved in corn oil) for 7 consecutive days (+ Niclo). Littermate F508del-cftr/F508del-cftr animals received corn oil only (−Niclo, control). Injections with Niclo were well tolerated and weight gain was normal. Animals were humanely killed and the intestine was stained for the presence of mucus using periodic acid*–*Schiff (PAS) reaction or alcian blue. When compared to wild type mice, enhanced PAS staining was found in the lumen of CF jejunum, which was attenuated in Niclo-treated animals (Fig. 5A,B). Quantitative analysis of alcian blue stained mucus showed significant inhibition of mucus production by intraperitoneal application of Niclo (Fig. 5C,D). Although less obvious, mucus staining by alcian blue was also reduced by Niclo in CF colon (Fig. 5E). Infiltration of colon by CD45 positive leukocytes was analysed and was found to be enhanced in F508del/F508del-cftr mice when compared to wild type mice.

**Figure 6:**
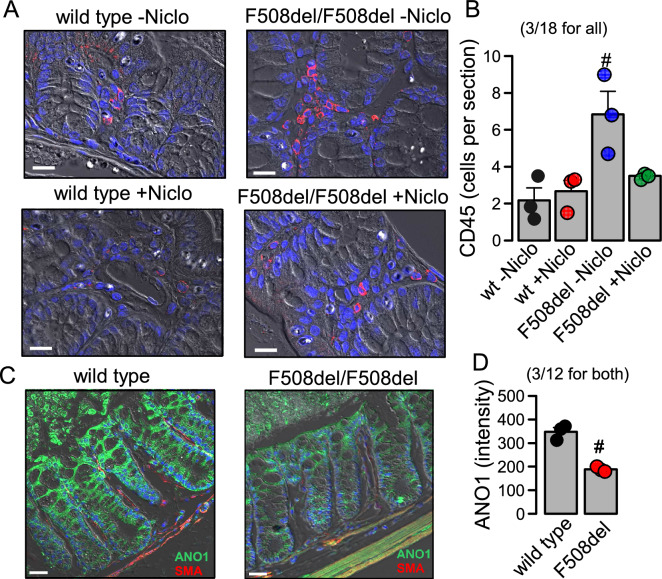
CD45 positive cells and expression of ANO1 in colon of wild type and F508del/F508del-cftr mice. (**A**) CD45 staining of leukocytes (red fluorescence) in wild type and F508del/F508del-cftr colon in the absence or presence niclosamide treatment (Niclo). (**B**) Summary of the number of CD45-positive cells per section. (n = 3 animals/n = 18 sections analysed for each animal). (**C**) Immunostaining of ANO1 (green) and smooth muscle actin (SMA; red) in colon of wild type and F508del/F508del-cftr mice. Nuclei are stained by Hoe33342. (**D**) Summary of ANO1 expression (intensity of green fluorescence). (n = 3 animals/n = 12 sections analysed for each animal). Mean ± SEM (number of animals/number of sections analysed). ^#^Significant difference when compared to wild type (p < 0.05; unpaired t-test).

Niclo treatment significantly reduced leukocyte infiltration, suggesting an anti-inflammatory effect of niclosamide, as observed previously in mouse airways^22^ (Fig. 6A,B). We also compared expression of ANO1 in wt and CF colon and detected weaker expression in CF (Fig. 6C,D). This finding could be related to the altered bronchial repair observed in CF, due to epithelial dedifferentiation^45,46^. In summary, these data show for the first time that niclosamide inhibits intestinal obstruction and inflammation in vivo in animals carrying the most common CFTR-mutation. It may therefore resemble a drug that is potentially useful in the treatment of cystic fibrosis.

### Inhibition of intestinal mucus secretion by benzbromarone in homozygous F508del-cftr rats

**Figure 7:**
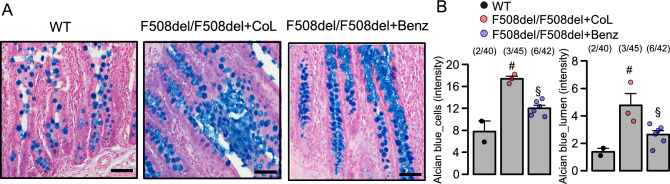
Inhibition of mucus secretion by benzbromarone in F508del/F508del-cftr rat jejunum. (**A**) Alcian blue staining of mucus in jejunum from a wild type rat, a rat homozygous for F508del-cftr maintained on ColonLytely (CoL), and a homozygous F508del-cftr rat treated with benzbromarone (Benz; 5 mg/kg body weight in drinking water for 60 days). Tissue staining by haematoxylin & eosin. (**B**) Summary of alcian blue intensity in cells (WT 2 animals/40 sections analysed; F508del + CoL 3 animals/45 sections analysed; F508del + Benz 6 animals/42 sections analysed) and lumen of jejunum (WT 2 animals/40 sections analysed; F508del + CoL 3 animals/45 sections analysed; F508del + Benz 6 animals/42 sections analysed). Mean ± SEM (number of animals/number of sections analysed). ^#^significant difference when compared to WT (p < 0.05; ANOVA & post hoc Bonferroni). ^§^significant difference when compared to F508del/F508del + CoL (p < 0.05; ANOVA & post hoc Bonferroni).

A rat model for cystic fibrosis was reported recently^43^. Similar to homozygous F508del-cftr mice, homozygous F508del-cftr rats demonstrate a mild lung phenotype, but exhibit mucus hypersecretion in small intestine^43^ (Fig. 7A). These CF rats required permanent oral supply of ColonLytely (CoL) in order to avoid intestinal obstructions and death^43^. We examined whether oral application of Benz reduces mucus secretion in small and large intestine of F508del-cftr rats. In fact, when Benz was provided with the feed for 60 days, CF rats could be maintained without oral supply of CoL, and mucus accumulation in jejunum was reduced (Fig. 7A,B).

**Figure 8:**
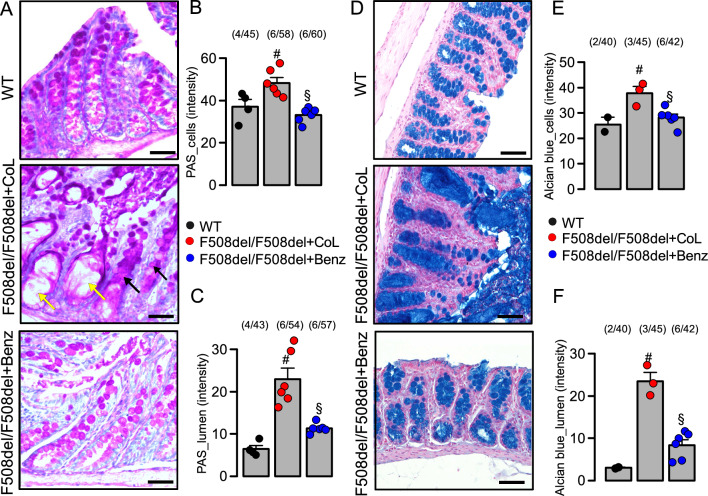
Inhibition of mucus secretion by benzbromarone in F508del/F508del-cftr rat colon. (**A**) PAS staining of mucus in colonic crypts of a wild type rat (WT), and rats homozygous for F508del-cftr maintained on ColonLytely (CoL) or treated with benzbromarone (Benz; 5 mg/kg body weight in drinking water for 60 days). (**B**,**C**) PAS intensity in intestinal crypt cells (black arrows) (WT 4 animals/45 sections analysed; F508del + CoL 6 animals/58 sections analysed; F508del + Benz 6 animals/60 sections analysed) and in extended crypt lumen (yellow arrow) (WT 4 animals/43 sections analysed; F508del + CoL 6 animals/54 sections analysed; F508del + Benz 6 animals/57 sections analysed). (**D**) Alcian blue staining of mucus in colonic crypts (WT 2 animals/40 sections analysed; F508del + CoL 3 animals/45 sections analysed; F508del + Benz 6 animals/42 sections analysed) and lumen (WT 2 animals/40 sections analysed; F508del + CoL 3 animals/45 sections analysed; F508del + Benz 6 animals/42 sections analysed) of a wild type rat (WT), and F508del/F508del-cftr rats maintained on CoL or treated with Benz. Tissue staining by haematoxylin & eosin. Mean ± SEM (number of animals/number of sections analysed). ^#^significant difference when compared to WT (p < 0.05; ANOVA & post hoc Bonferroni). ^§^significant difference when compared to F508del/F508del + CoL (p < 0.05; ANOVA & post hoc Bonferroni).

Analysis of mucus secretion in the large intestine by PAS (Fig. 8A–C) or alcian blue (Fig. 8D–F) also revealed enhanced mucus secretion and crypt dilation in CF rats, which was normalized by treatment with Benz. In summary, these data suggest that augmented intestinal mucus production in CF rats and mice can be alleviated by benzbromarone and niclosamide, which both may prevent fatal gut obstructions. We conclude that niclosamide and benzbromarone may have the potential to improve animal welfare and are suggested as potential treatments for cystic fibrosis gut and lung disease.

## Discussion

The present results demonstrate reduced mucus secretion in airways and intestine by the common drugs niclosamide and benzbromarone, which is probably due to inhibition ANO1 and intracellular Ca^2+^ signals. Constitutive mucus secretion as well as mucus secretion elicited by secretagogues is driven by the Cl^−^ channel ANO1 and the phospholipid scramblase and ion channel ANO6, which are both highly expressed in goblet cells of airways and intestine. During inflammation, ANO1 and ANO6 are upregulated^4,47^. ANO1 has been shown to upregulate intracellular submembraneous Ca^2+^ levels which probably activates Ca^2+^-sensitive proteins of the exocytic machinery, such as synaptotagmin and Munc13^7,24,48^. ANO1 also supports Ca^2+^-dependent activation of ANO6, which was shown to support exocytosis and membrane shedding^49,50^.

The drugs were applied at low concentrations, typically present in the plasma of treated patients. At ≥ 100 nM niclosamide caused a biphasic effect on intracellular [Ca^2+^]_i_, which was also found in other studies^51,52^. The authors of these reports argued that niclosamide does not block ANO1 but rather inhibits ANO1 indirectly by altering intracellular Ca^2+^ signals^26,27^. However, in the present study ANO1 was also inhibited when activated by a Ca^2+^ ionophore or by 1 µM Ca^2+^ in the patch pipette, clearly suggesting a direct inhibition of ANO1 (Fig. 1). Nevertheless, the present and previous data also demonstrate reduced basal [Ca^2+^]_i_ and inhibition of ATP-induced Ca^2+^ store release by ANO1 blockers such as niflumic acid, CaCC_inh_-AO1^28^ or niclosamide. These ANO1 inhibitors seem to cause a reduced Ca^2+^ uptake into the ER, and thus lower ER store content^22,23,25^. Notably, niclosamide had little effect on [Ca^2+^]_i_ when ANO1 expression was knocked down^25^. Moreover, in mice suffering from polycystic kidney disease, ANO1 expression and Ca^2+^ signals were found to be upregulated, and both was reversed with knockout of ANO1^53^. These and other data indicate that ANO1 expression and function controls [Ca^2+^]_i_^28^.

ANO1 augments compartmentalized Ca^2+^ signals by its ability to bind to IP_3_ receptors, which are ER Ca^2+^ release channels (IP_3_R). Binding of ANO1 to IP_3_R tethers the ER near the plasma membrane^28,34^. This facilitates binding of IP_3_ to IP_3_R and augments Ca^2+^ release. Moreover, activation of ANO1 may enhance the activity of IP_3_R, thereby augmenting store release. These effects are suppressed in the presence of ANO1 inhibitors^25^. As Ca^2+^ influx channels such as TRPV1 and TRPV4 also interact with ANO1, it is likely that Ca^2+^ influx is shaped by expression and activity of ANO1^35–38^. TRPV4 channels are well expressed in CFBE airway epithelial cells, along with the ryanodine receptor RyR2, which lead to Ca^2+^ store release when the membrane voltage is depolarized due to activation of ANO1 and efflux of Cl^−^ ions^54^ (Fig. S1).

The present data demonstrate inhibition of mucus production/secretion by niclosamide and benzbromarone in airways and gut, yet the described effects on intracellular Ca^2+^ may prevent their use in asthma and CF, or other diseases such as COVID-19, polycystic kidney disease, or cancer^55^. It should be considered that niclosamide is on the WHO list of essential medicines and is very well tolerated when applied orally at the standard dose of 2 g/day, which leads to plasma concentrations used in the present study^30^. Recent clinical studies show that local airway application of even very large concentrations of niclosamide is well tolerated in human and mouse^4,23,56–58^. Benzbromarone has been used for the treatment of gout for more than 30 years. The reported hepatotoxicity is rare and occurs only in 1:17,000 patients^59^, and thus withdrawal of benzbromarone from the market has been questioned^59^. It was well tolerated when used in a recently finished pilot clinical trial with CF patients^60^. (iii) Treatment of mice with the ANO inhibitors niclosamide, benzbromarone or Ani9, a more specific inhibitor of ANO1, was highly effective in reducing renal cyst growth and did not cause any obvious side effects^53^. It may therefore be worthwhile to pursue these compounds in future clinical trials.

We conclude that well tolerated therapeutic concentrations of niclosamide and benzbromarone that correspond to plasma levels of treated patients, inhibit ANO1 and intracellular Ca^2+^ signals. The drugs may be useful in inhibiting mucus hypersecretion and mucus obstruction in airways and intestine of patients suffering from asthma and CF, respectively. Moreover, the present results show for the first time an inhibition of intestinal mucus secretion and inflammatory symptoms in vivo in two independent animal models for CF. To our knowledge this is the first time that benzbromarone was applied to inflamed (asthmatic) airways in vivo, and a muco-inhibitory effect comparable to that of niclosamide was found. Because these compounds will reduce pulmonary and intestinal mucus load regardless of the underlying disease, they may be generally useful in the treatment of muco-obstructive diseases.

## Methods

### Animals

All animal experiments complied with the guidelines for animal research and were carried out in accordance with the ‘United Kingdom Animals Act, 1986’ and associated guidelines, as well as EU Directive 2010/63/EU for animal experiments. All animal experiments were approved by the local Ethics Committee of the Government of Unterfranken/Wurzburg (AZ: 55.2.2-2532-2-1359-15), the University of Adelaide (M-2019-034), or by the animal welfare committee of Hannover Medical School and the Landesamt für Verbraucherschutz und Lebensmittelsicherhet, Abtl. Veterinärininstitut (LAVES) for Lower Saxony (Az. 33.14-42502-04-14/1549 and Az 33.12-42502-04-19/3197) Breeding “stressed strains”, were conducted according to the guidelines of the American Physiologic Society, German Law for the Welfare of Animals or the National Health and Medical Research Council (Australia), and adhered to the ARRIVE guidelines.

### Niclosamide and benzbromarone treatment of asthmatic mice

Ovalbumin sensitization has been described earlier^61^. In brief, Mice were sensitized to OVA (MilliporeSigma) by intraperitoneal (i.p.) injection of 100 μg OVA with 1 mg aluminium hydroxide gel (MilliporeSigma) on days 0 and 14. On days 21, 22, and 23, mice were anesthetized and challenged to OVA by intratracheal instillation (IT) of 50 μg OVA in 100 μL saline. Control mice were sham sensitized with saline and aluminium hydroxide gel (MilliporeSigma) and challenged by 100 μL saline IT. 30 µM niclosamide (Niclo) or 30 µM benzbromarone (Benz) were dissolved in 100 µl saline and were applied IT for 5 consecutive days. 100 µl saline only (OVA) served as control. We choose a 30 µM concentration, because in a recent phase 1 clinical trial a similar concentration of niclosamide was directly applied to human airways, which was well tolerated^58^. Although this dose is high, one should consider that only a fraction of the drugs will reach airway epithelial cells, as they need to penetrate the airway mucus layer and will be also flushed away by the mucociliary clearance.

Generation, maintenance and characterisation of the CF mouse colony has been described in previous studies^62,63^. In brief, the homozygous F508del-cftr mouse strain was maintained on low fibre enhanced fat diet and received a polyethylene glycol-containing laxative drinking solution as cited above. Mouse airways were fixed by transcardial perfusion and lung perfusion by tracheal instillation via tracheostomy of fixative solution containing 4% PFA in PBS. Tissues were left in fixative solution overnight and embedded in paraffin the next day. Mouse intestines were fixed by perfusion with 4% paraformaldehyde (PFA) and post-fixed in 0.5 mol/l sucrose, 4% PFA solution. 5 µm sections were deparaffinized, stained with standard Alcian blue solution, and counterstained with Nuclear Fast Red solution (Sigma-Aldrich, St. Louis, MO, USA). Quantification of mucus stained by alcian blue or PAS was performed as recently by transferring images to the analysis program ImageJ FIJI version 1.53e (National Institute of Health and the Laboratory for Optical and Computational Instrumentation (LOCI, University of Wisconsin)^23,64^. Numbers form intensity readings were used for further statistical analysis.

### Niclosamide treatment of F508del-cftr rats

Generation, maintenance and characterisation of the F508del-cftr rat colony has been detailed in a previous paper^43^. The homozygous F508del-cftr rats were maintained on a 50:50 mix of normal (6.5%) and high-fat (9%) irradiated rodent chow (Envigo 2920X and 2919, Indianapolis, USA) and 4.5% ColonLytely (Dendy Pharmaceuticals, Australia) in drinking water. Initially a test run was performed using wildtype rats (n = 5) to validate that benzbromarone (5 mg/kg body weight) in jelly (Aeroplane Original Strawberry flavoured, Australia) was sufficiently palatable that the animals will consume it. Jelly was made up at per packet instruction; 10.6 g of jelly powder, 31.25 mL boiling water, and 25 mL cold water. Once the jelly cooled and began to solidify, 31.25 mg of benzbromarone was mixed into the 62.5 g jelly. Rats were fed a dose of 5 mg benzbromarone/kg body weight/day. Five adult CF rats were switched to receive benzbromarone and ColonLytely in drinking water as a combined therapy, which did not cause adverse reactions. CF rats were weaned onto benzbromarone only, without receiving ColonLytely (F508del/F508del + Benz; n = 8). Due to animal welfare concerns we did not include a no-treatment (i.e. water only) CF animal group, as these animals would likely suffer rapid fatal gut obstructions. Instead, CF rats fed with ColonLytely without benzbromarone served as controls (F508del/F508del + CoL; n = 6). Weight gain of all animals was assessed daily, and all animals were carefully monitored for the development of symptoms of intestinal blockage. After 60 days, animals were killed by CO_2_ asphyxiation and the intestine was removed for macroscopic inspection, fixed in 10% formalin for at least 24 h and was embedded in paraffin.

### Immunohistochemistry

Five µm sections were deparaffinized and incubated afterwards with primary ANO1-antibody in 0.5% BSA and 0.04% Triton X-100 overnight at 4 °C, followed by incubation with Alexa Fluor 488 labelled donkey anti-rabbit IgG (Invitrogen). Affinity-purified polyclonal antiserum against mouse Ano1 was produced in rabbits immunized with (mouse) NHSPTTHPEAGDGSPVPSYE (aa957-976, C-terminus) coupled to keyhole limpet hemocyanin (Davids Biotechnologie, Regensburg, Germany). Infiltration by immune cells was assessed using the pan-leukocyte marker CD45. Sections were counterstained with Hoe33342 (Sigma-Aldrich) for nuclei visualisation. Immunofluorescence was detected using an Axiovert 200 microscope equipped with ApoTome and Axio-Vision (Zeiss, Germany).

### Cells

Culture conditions for the CFBE41o- cell line has been described earlier^22^. In brief, CFBE41o- cells^65^ were grown in MEM with Earle’s Salts and l-Glutamine medium (Capricorn Scientific, Ebsdorfergrund, Germany) supplemented with 10% fetal bovine serum. Cells were grown at 37 °C in the absence of antibiotics in a humidified atmosphere with 5% CO_2_. For experiments, cells were grown on glass coverslips and subsequently mounted into a perfused bath on the stage of an inverted microscope (Zeiss, Axiovert 200).

### Ca^2+^ measurements

Measurement of cytosolic Ca^2+^ changes were performed as described recently^66^. In brief, CFBE cells were loaded with 2 μM Fura2-AM (BIOZOL, Eching, Germany) in OptiMEM (Gibco, Thermo Fisher Scientific) with 0.02% Pluronic F-127 (Invitrogen, Thermo Fisher Scientific,) in ringer solution (mmol/l: NaCl 145; KH_2_PO_4_ 0.4; K_2_HPO_4_ 1.6; Glucose 5; MgCl_2_ 1; Ca^2+^-Gluconat 1.3) for 1 h at room temperature. Fluorescence was detected in cells perfused with Ringer's solution at 37 °C using an inverted microscope (Axiovert S100, Zeiss, Germany) and a high-speed polychromator system (VisiChrome, Puchheim, Germany). Fura2 was excited at 340/380 nm, and the emission was recorded between 470 and 550 nm using a CCD camera (CoolSnap HQ, Visitron Systems, Germany). [Ca^2+^]i was calculated from the 340/380 nm fluorescence ratio after background subtraction. The formula used to calculate [Ca^2+^]i was [Ca^2+^]i = Kd × (R − Rmin)/(Rmax − R) × (Sf2/Sb2), where R is the observed fluorescence ratio. The values Rmax and Rmin (maximum and minimum ratios) and the constant Sf2/Sb2 (fluorescence of free and Ca^2+^-bound Fura-2 at 380 nm) were calculated using 2 μmol/L ionomycin (Biomol GmbH, Hamburg, Germany) and 5 mmol/L EGTA to equilibrate intracellular and extracellular Ca^2+^ in intact Fura-2-loaded cells. The dissociation constant for the Fura-2•Ca^2+^ complex was taken as 224 nmol/L^67^. Control of experiment, imaging acquisition, and data analysis were done with the software package Meta- Fluor (Universal imaging, USA). When using the niclosamide or benzbromarone, inhibitors were applied 5 min before starting the experiment. They were present throughout the experiment.

### Patch clamp

Cells used for patch clamp experiments were grown on coated glass coverslips. Coverslips were mounted in a perfused bath chamber on the stage of an inverted microscope (IM35, Zeiss) and kept at 37 °C. Patch pipettes were filled with a cytosolic-like solution containing (in mM): KCl 30, K-Gluconate 95, NaH_2_PO_4_ 1.2, Na_2_HPO_4_ 4.8, EGTA 1, Ca-Gluconate 0.758, MgCl_2_ 1.03, d-Glucose 5, ATP 3; pH 7.2. The intracellular (pipette) Ca^2+^ activity was 0.1 μM. The bath was perfused continuously with standard bicarbonate-free Ringer's solution (in mM: NaCl 145, KH_2_PO_4_ 0.4, K_2_HPO_4_ 1.6, Glucose 5, MgCl_2_ 1, Ca^2+^—Gluconate 1.3) at a rate of 4 ml/min. Patch pipettes had an input resistance of 3–5 MΩ and whole-cell currents were corrected for serial resistance. Currents were recorded using a patch clamp amplifier EPC9, and PULSE software (HEKA, Lambrecht, Germany) as well as Chart software (AD Instruments, Spechbach, Germany). Cells were stimulated with 1 μM ATP in the absence and presence of TRAM34. In regular intervals, membrane voltage (Vc) was clamped in steps of 20 mV from − 100 to + 100 mV from a holding voltage of − 100 mV. The current density was calculated by dividing whole- cell currents by cell capacitance.

### Materials and statistics

All compounds used were of highest available grade of purity and were purchased from Sigma/Aldrich (Deisenhofen, Germany). Data are reported as mean ± SEM. Student’s t test (for unpaired samples) or one-way ANOVA was used for statistical analysis. A P value less than 0.05 was accepted as a significant difference.

### Supplementary Information

Supplementary Figure S1.

Supplementary Legends.

Supplementary Information.

## Data Availability

Original data and materials are available on request. Please contact Prof. Dr. Karl Kunzelmann (karl.kunzelmann@ur.de).

## References

[1] Huang F (2012). Calcium-activated chloride channel TMEM16A modulates mucin secretion and airway smooth muscle contraction. Proc. Natl. Acad. Sci. USA.

[2] Zhang CH (2013). The transmembrane protein 16A Ca(2+)-activated Cl^−^ channel in airway smooth muscle contributes to airway hyperresponsiveness. Am. J. Respir. Crit. Care Med..

[3] Caci E (2015). Upregulation of TMEM16A protein in bronchial epithelial cells by bacterial pyocyanin. PLoS ONE.

[4] Centeio R (2021). Mucus release and airway constriction by TMEM16A May Worsen pathology in inflammatory lung disease. Int. J. Mol. Sci..

[5] Scudieri P (2012). Association of TMEM16A chloride channel overexpression with airway goblet cells metaplasia. J. Physiol..

[6] Lin J (2015). TMEM16A mediates the hypersecretion of mucus induced by Interleukin-13. Exp. Cell Res..

[7] Benedetto R, Cabrita I, Schreiber R, Kunzelmann K (2019). TMEM16A is indispensable for basal mucus secretion in airways and intestine. FASEB J..

[8] Cabrita I (2020). TMEM16A mediated mucus production in human airway epithelial cells. Am. J. Respir. Cell Mol. Biol..

[9] Centeio R, Ousingsawat J, Schreiber R, Kunzelmann K (2021). CLCA1 regulates airway mucus production and ion secretion through TMEM16A. Int. J. Mol. Sci..

[10] Qin Y (2016). Interleukin-13 stimulates MUC5AC expression via a STAT6-TMEM16A-ERK1/2 pathway in human airway epithelial cells. Int. Immunopharmacol..

[11] Kondo M (2017). Chloride ion transport and overexpression of TMEM16A in a guinea pig asthma model. Clin. Exp. Allergy.

[12] Danielsson J (2015). Antagonists of the TMEM16A calcium-activated chloride channel modulate airway smooth muscle tone and intracellular calcium. Anesthesiology.

[13] Miner K (2019). Drug repurposing: The anthelmintics niclosamide and nitazoxanide are potent TMEM16A antagonists that fully bronchodilate airways. Front. Pharmacol..

[14] Danielsson J (2020). Agonism of the TMEM16A calcium-activated chloride channel modulates airway smooth muscle tone. Am. J. Physiol. Lung Cell Mol. Physiol..

[15] Forrest AS (2012). Increased TMEM16A-encoded calcium-activated chloride channel activity is associated with pulmonary hypertension. Am. J. Physiol. Cell Physiol..

[16] Allawzi AM (2018). Activation of anoctamin-1 limits pulmonary endothelial cell proliferation via p38-mitogen-activated protein kinase-dependent apoptosis. Am. J. Respir. Cell Mol. Biol..

[17] Papp R (2019). Targeting TMEM16A to reverse vasoconstriction and remodelling in idiopathic PAH. Eur. Respir. J..

[18] Liu D (2020). TMEM16A regulates pulmonary arterial smooth muscle cells proliferation via p38MAPK/ERK pathway in high pulmonary blood flow-induced pulmonary arterial hypertension. J. Vasc. Res..

[19] Galietta LJV (2022). TMEM16A (ANO1) as a therapeutic target in cystic fibrosis. Curr. Opin. Pharmacol..

[20] Kunzelmann K (2019). TMEM16A in cystic fibrosis: Activating or inhibiting?. Front. Pharmacol..

[21] Feng S (2023). Identification of a drug binding pocket in TMEM16F calcium-activated ion channel and lipid scramblase. Nat. Commun..

[22] Cabrita I, Benedetto R, Schreiber R, Kunzelmann K (2019). Niclosamide repurposed for the treatment of inflammatory airway disease. JCI Insight.

[23] Ousingsawat J (2022). Airway delivery of hydrogel-encapsulated niclosamide for the treatment of inflammatory airway disease. Int. J. Mol. Sci..

[24] Centeio R, Cabrita I, Schreiber R, Kunzelmann K (2023). TMEM16A/F support exocytosis but do not inhibit Notch-mediated goblet cell metaplasia of BCi-NS1.1 human airway epithelium. Front. Physiol..

[25] Centeio R (2020). Pharmacological inhibition and activation of the Ca(2+) activated Cl(−) channel TMEM16A. Int. J. Mol. Sci..

[26] Genovese M (2022). Analysis of a panel of TMEM16A chloride channel inhibitors reveals indirect mechanisms involving alteration of calcium signaling. Br. J. Pharmacol..

[27] Danahay H (2023). Niclosamide does not modulate airway epithelial function through blocking of the calcium activated chloride channel, TMEM16A. Front. Pharmacol..

[28] Cabrita I (2017). Differential effects of anoctamins on intracellular calcium signals. FASEB J..

[29] Andrews P, Thyssen J, Lorke D (1982). The biology and toxicology of molluscicides, Bayluscide. Pharmacol. Ther..

[30] Singh S (2022). Niclosamide-A promising treatment for COVID-19. Br. J. Pharmacol..

[31] Uchida S (2010). Benzbromarone pharmacokinetics and pharmacodynamics in different cytochrome P450 2C9 genotypes. Drug Metab. Pharmacokinet..

[32] Benedetto R (2017). Epithelial chloride transport by CFTR requires TMEM16A. Sci. Rep..

[33] Schreiber R, Faria D, Skryabin BV, Rock JR, Kunzelmann K (2015). Anoctamins support calcium-dependent chloride secretion by facilitating calcium signaling in adult mouse intestine. Pflügers Arch..

[34] Jin X (2013). Activation of the Cl^−^ channel ANO1 by localized calcium signals in nociceptive sensory neurons requires coupling with the IP3 receptor. Sci. Signal.

[35] Zhang Y (2022). Functional coupling between TRPV4 channel and TMEM16F modulates human trophoblast fusion. Elife.

[36] Shah S (2020). Local Ca(2+) signals couple activation of TRPV1 and ANO1 sensory ion channels. Sci. Signal..

[37] Takayama Y, Shibasaki K, Suzuki Y, Yamanaka A, Tominaga M (2014). Modulation of water efflux through functional interaction between TRPV4 and TMEM16A/anoctamin 1. FASEB J..

[38] Takayama Y, Uta D, Furue H, Tominaga M (2015). Pain-enhancing mechanism through interaction between TRPV1 and anoctamin 1 in sensory neurons. Proc. Natl. Acad. Sci. USA.

[39] Schiebler ML (2023). Imaging regional airway involvement of asthma: Heterogeneity in ventilation, mucus plugs and remodeling. Adv. Exp. Med. Biol..

[40] Mall MA (2016). Unplugging mucus in cystic fibrosis and chronic obstructive pulmonary disease. Ann. Am. Thorac. Soc..

[41] Parmley RR, Gendler SJ (1998). Cystic fibrosis mice lacking Muc1 have reduced amounts of intestinal mucus. J. Clin. Investig..

[42] McCarron A, Donnelley M, Parsons D (2018). Airway disease phenotypes in animal models of cystic fibrosis. Respir. Res..

[43] McCarron A (2020). Phenotypic characterization and comparison of cystic fibrosis rat models generated using CRISPR/Cas9 gene editing. Am. J. Pathol..

[44] Garcia MA, Yang N, Quinton PM (2009). Normal mouse intestinal mucus release requires cystic fibrosis transmembrane regulator-dependent bicarbonate secretion. J. Clin. Investig..

[45] Ruffin M (2013). Anoctamin 1 dysregulation alters bronchial epithelial repair in cystic fibrosis. Biochim. Biophys. Acta.

[46] Salari A (2023). The anion channel TMEM16a/Ano1 modulates CFTR activity, but does not function as an apical anion channel in colonic epithelium from cystic fibrosis patients and healthy individuals. Int. J. Mol. Sci..

[47] Schreiber R, Cabrita I, Kunzelmann K (2022). Paneth cell secretion in vivo requires expression of Tmem16a and Tmem16f. Gastro Hep. Adv..

[48] Ousingsawat, J., Centeio, R., Schreiber, R. & Kunzelmann, K. Niclosamide, but not ivermectin, inhibits anoctamin 1 and 6 and attenuates inflammation of the respiratory tract. *Pflugers Arch.* in press (2023). 10.1007/s00424-023-02878-w PMC10791962 37979051

[49] Ousingsawat J, Schreiber R, Kunzelmann K (2019). TMEM16F/anoctamin 6 in ferroptotic cell death. Cancers.

[50] Sommer A (2016). Phosphatidylserine exposure is required for ADAM17 sheddase function. Nat. Commun..

[51] Liang, P., Wan, Y. C. S., Yu, K., Hartzell, H. C. & Yang, H. Niclosamide potentiates TMEM16A and induces vasoconstriction. *bioRxiv preprint* (2023). 10.1085/jgp.202313460 PMC11138202 38814250

[52] Dwivedi R (2023). The TMEM16A blockers benzbromarone and MONNA cause intracellular Ca(2+)-release in mouse bronchial smooth muscle cells. Eur. J. Pharmacol..

[53] Cabrita I (2020). Cyst growth in ADPKD is prevented by pharmacological and genetic inhibition of TMEM16A in vivo. Nat. Commun..

[54] Liu QH (2009). Membrane depolarization causes a direct activation of G protein-coupled receptors leading to local Ca^2+^ release in smooth muscle. Proc. Natl. Acad. Sci. USA.

[55] Wang Z (2022). Niclosamide as a promising therapeutic player in human cancer and other diseases. Int. J. Mol. Sci..

[56] Cairns DM (2022). Efficacy of niclosamide vs placebo in SARS-CoV-2 respiratory viral clearance, viral shedding, and duration of symptoms among patients with mild to moderate COVID-19: A phase 2 randomized clinical trial. JAMA Netw. Open.

[57] Jiang H, Li AM, Ye J (2022). The magic bullet: Niclosamide. Front. Oncol..

[58] Backer V (2021). A randomized, double-blind, placebo-controlled phase 1 trial of inhaled and intranasal niclosamide: A broad spectrum antiviral candidate for treatment of COVID-19. Lancet Reg. Health. Eur..

[59] Lee MH, Graham GG, Williams KM, Day RO (2008). A benefit-risk assessment of benzbromarone in the treatment of gout. Was its withdrawal from the market in the best interest of patients?. Drug Saf..

[60] Friedrich FF (2022). Benzbromarone for the treatment of cystic fibrosis (CF) lung disease: A pilot clinical trial. Eur. Respir. J..

[61] Schreiber R, Castrop H, Kunzelmann K (2008). Allergen induced airway hyperresponsiveness is absent in ecto-5′-nucleotidase (CD73) deficient mice. Pflugers Arch..

[62] Xiao F (2012). Rescue of epithelial HCO_3_^−^ secretion in murine intestine by apical membrane expression of the cystic fibrosis transmembrane conductance regulator mutant F508del. J. Physiol..

[63] Tan Q (2021). Inhibition of Na(+)/H(+) exchanger isoform 3 improves gut fluidity and alkalinity in cystic fibrosis transmembrane conductance regulator-deficient and F508del mutant mice. Br. J. Pharmacol..

[64] Schindelin J (2012). Fiji: An open-source platform for biological-image analysis. Nat. Methods.

[65] Hentchel-Franks K (2004). Activation of airway Cl^−^ secretion in human subjects by adenosine. Am. J. Respir. Cell Mol. Biol..

[66] Cabrita I, Talbi K, Kunzelmann K, Schreiber R (2021). Loss of PKD1 and PKD2 share common effects on intracellular Ca^2+^ signaling. Cell Calcium.

[67] Grynkiewicz G, Poenie M, Tsien RY (1985). A new generation of Ca^2+^ indicators with greatly improved fluorescence properties. J. Biol. Chem..

