# Supplementary figures and images for: Inhibition of mucus secretion by niclosamide and benzbromarone in airways and intestine
Source: Sci Rep. 2024 Jan 17;14:1464. doi: 10.1038/s41598-024-51397-w (PMC10794189; doi:10.1038/s41598-024-51397-w)

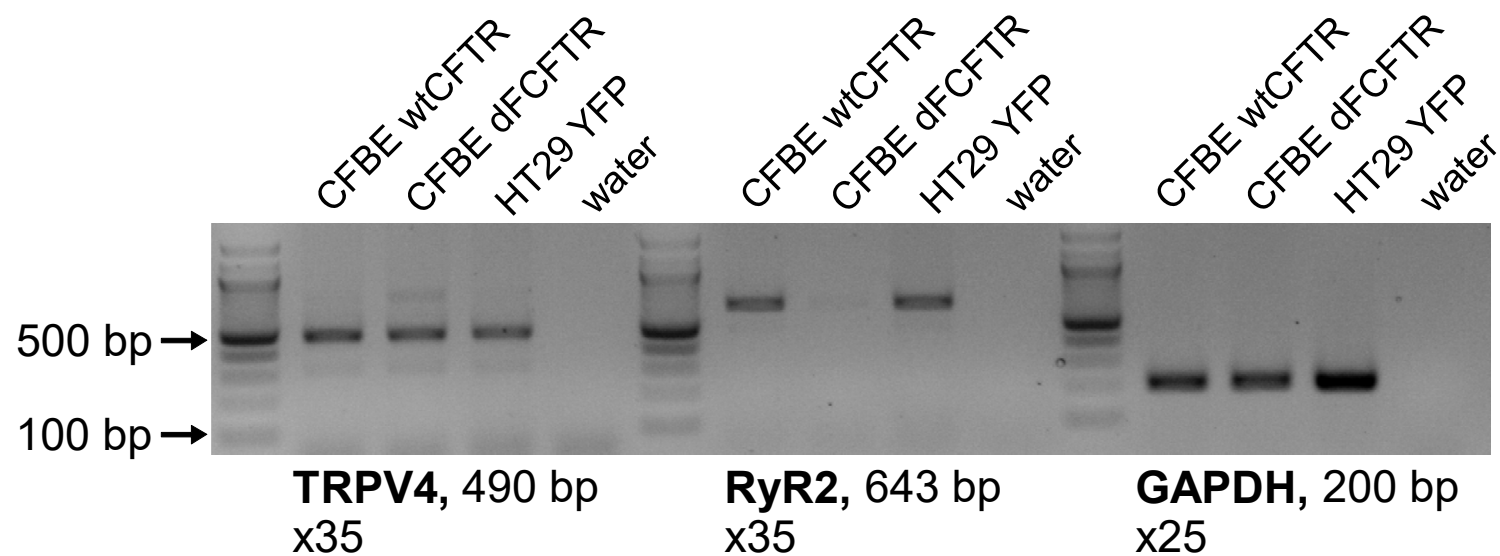

Supplement: Supplementary file 1 — Supplementary Figure S1. [file 41598_2024_51397_MOESM1_ESM.pdf]

# Uncropped blots

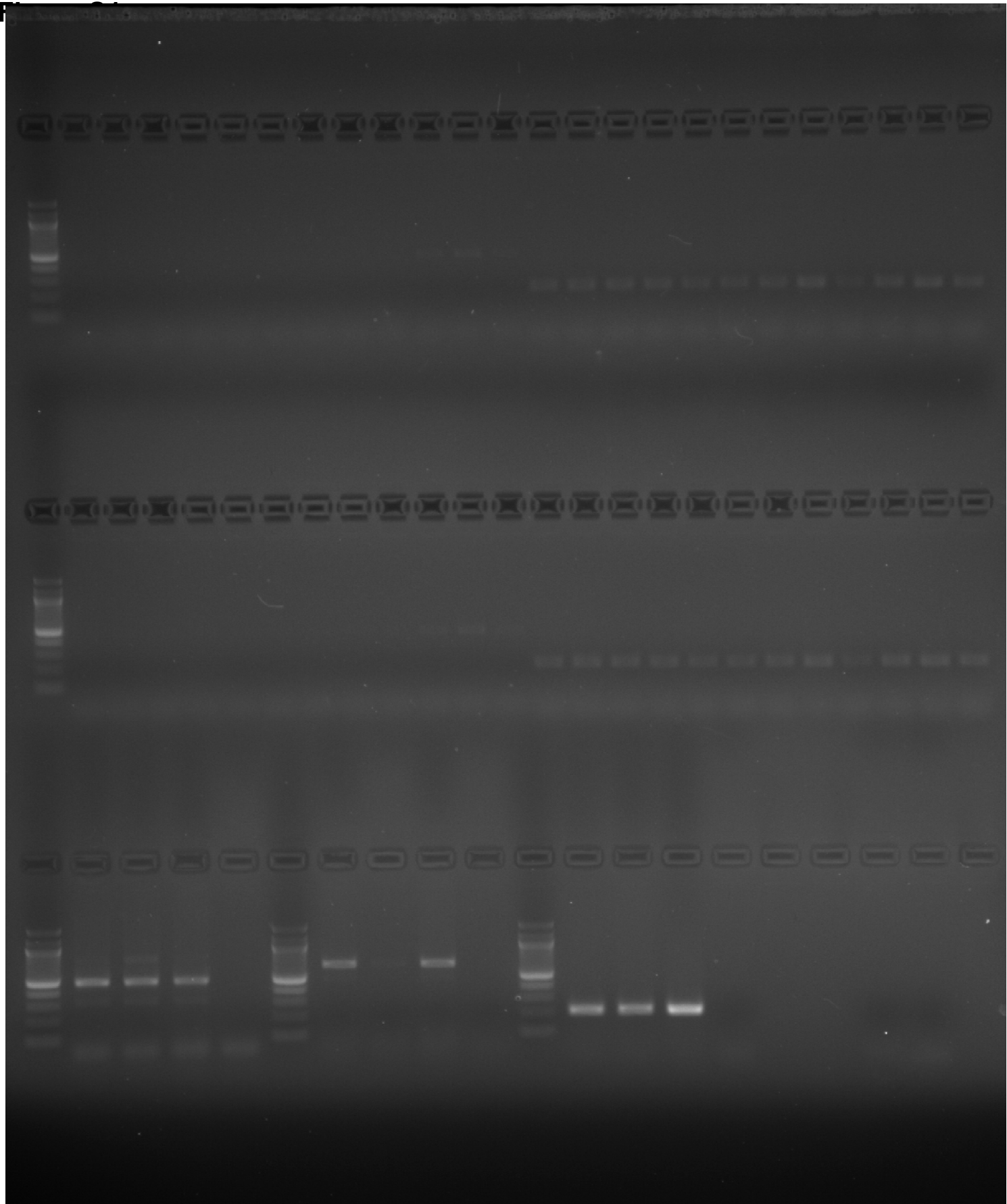

Supplement: Supplementary file 3 — Supplementary Information. [file 41598_2024_51397_MOESM3_ESM.pdf]
