# Supplementary material for: Inhibition of mucus secretion by niclosamide and benzbromarone in airways and intestine
Source: Sci Rep. 2024 Jan 17;14:1464. doi: 10.1038/s41598-024-51397-w (PMC10794189; doi:10.1038/s41598-024-51397-w)
Supplement: Supplementary file 2 — Supplementary Legends. [file 41598_2024_51397_MOESM2_ESM.pdf]

Supplementary Figure 1. *Expression of TRPV4 and RyR2 in CFBE cells.* RT-PCR of TRPV4 and RyR2 in CFBE airway epithelial cells and HT_{29} colonic epithelial cells.
